# Rural youths' understanding of gene x environmental contributors to heritable health conditions: The case of podoconiosis in Ethiopia

**DOI:** 10.1371/journal.pntd.0006763

**Published:** 2018-09-13

**Authors:** Kibur Engdawork, Colleen M. McBride, Desta Ayode, Caitlin G. Allen, Gail Davey, Getnet Tadele

**Affiliations:** 1 Department of Sociology, College of Social Sciences, Addis Ababa University, Addis Ababa, Ethiopia; 2 Rollins School of Public Health, Emory University, Atlanta, GA, United States of America; 3 Brighton and Sussex Medical School, University of Sussex, Falmer, Brighton, United Kingdom; National Institutes of Health, UNITED STATES

## Abstract

**Objectives:**

Assess the feasibility of engaging youth to disseminate accurate information about gene by environmental (GxE) influences on podoconiosis, a neglected tropical lymphedema endemic in southern Ethiopia.

**Methods:**

A cross sectional survey was conducted with 377 youth randomly selected from 2 districts of Southern Ethiopia. Measures included GxE knowledge (4 true/false statements), preventive action knowledge (endorse wearing shoes and foot hygiene), causal misconceptions (11 items related to contagion) and confidence to explain GxE (9 disagree/agree statements).

**Results:**

Over half (59%) accurately endorsed joint contributions of gene and environment to podoconiosis and preventive mechanisms (e.g., wearing protective shoes and keeping foot hygiene). Multivariable logistic regression showed that youth with accurate understanding about GxE contributors reported having: some education, friends or kin who were affected by the condition, and prior interactions with health extension workers. Surprisingly, higher accurate GxE knowledge was positively associated with endorsing contagion as a causal factor. Accuracy of GxE and preventive action knowledge were positively associated with youth’s confidence to explain podoconiosis-related information.

**Conclusions:**

Youth have the potential to be competent disseminators of GxE information about podoconiosis. Interventions to foster confidence among youth in social or kin relationships with affected individuals may be most promising. Efforts to challenge youth’s co-existing inaccurate beliefs about contagion could strengthen the link of GxE explanations to preventive actions.

## Author summary

This study considers the feasibility of engaging rural Ethiopian youth as lay health workers (LHWs) with the objective to improve community understanding of the joint influences of genetics and environment on health. Identifying LHWs to accurately convey contributors to the heritable but preventable neglected tropical disease of podoconiosis provides an optimal context to address this question. Misunderstanding that inherited susceptibility to podoconiosis makes the disease unavoidable has led to numerous negative social consequences (e.g., stigma) and poor uptake of protective footwear. We report data from a pilot study that included a cross-sectional survey of youth ages 15–24 in rural communities with endemic podoconiosis. Results provide preliminary support that a sizeable group of youth hold accurate knowledge about gene x environment influences and self-evaluate as being confident to explain these associations to others. Research to evaluate strategies to engage youth as LHWs and the impact of these approaches on communities’ understanding of the joint influences of genetics and environment in this context is needed. This manuscript fills an important gap in the literature about neglected tropical diseases, as it suggests opportunities to improve the prevention of podoconiosis and reduce misconceptions and stigma through engagement of LHWs in Ethiopia.

## Introduction

Advances in genomics are increasing scientific understanding that most health conditions worldwide are caused by the joint influence of genetic and environmental (GxE) factors [1]. However, the mechanisms underlying GxE interactions are complex and not well understood by the public [2]. Accordingly, misunderstandings that health conditions with genetic underpinnings cannot be prevented have been well documented in the developing world [3–6]. Global leaders have called for stepping up efforts to increase genomic literacy in low and middle-income countries (LMICs) [7] [8, 9]. Among the challenges to achieving this imperative is that LMICs have limited health service infrastructure, low levels of general literacy and a majority of the populace lives in isolated rural settings. Funding opportunities such as the Human Heredity and Health in Africa (H3Africa) have been initiated to address these challenges [10].

Concurrent with advances in genomics, around the world, cadres of lay health workers (LHWs) have been engaged to expand the reach of preventive health services for a broad array of communicable and noncommunicable health conditions [11, 12]. LHWs carry out a number of functions to promote health including the provision of culturally relevant health information that can be conveyed in everyday community settings [13, 14]. Though it has been suggested that such LHWs might play an important role in promoting community-wide GxE literacy in the developed world to date there has been little consideration of this possibility in LMICs [15, 16] [17].

Efforts directed to disease prevention often target youth as a means to encourage the establishment of healthy behaviors. Considering youth as potential disseminators of information about GxE health influences offers several other advantages. As members of the community, youth are aware of existing interpretations of the causes of health conditions held by their peers and adults in the community [18]. In the context of HIV/AIDs and other STIs, reproductive health, and malaria, studies suggest that youth may more easily process and understand new information [3]. Youth also have been found to have greater accuracy in considering the influence of genes on health conditions than older adults [19]. These information-processing assets have been attributed to youth’s neurodevelopment stage, exposure to schools, youth networks and the media [20]. The feasibility of engaging LHWs to increase GxE literacy in LMICs rests on whether individuals can be identified with the capability and confidence to serve as GxE information disseminators.

Podoconiosis, a non-filarial lymphedema endemic in highland Ethiopia, offers an excellent context to consider the feasibility of engaging youth LHWs for promoting GxE literacy. Globally, it is estimated that four million people in tropical Africa, Central and South America, and Southeast Asia have the condition; approximately 1.5 million people are affected in Ethiopia [4]. The condition develops when genetically susceptible individuals are exposed to irritant particles in volcanic soil via walking and farming barefoot [21–23]. Podoconiosis can be prevented if susceptible individuals begin wearing shoes at an early age and do so consistently [21–23].

Prior health education efforts to promote shoe wearing among at-risk individuals have been based on extensive qualitative and rigorous quantitative evaluation of these interventions show that adults in these communities continue to harbor misconceptions that the condition is not preventable because of its heritability [5, 24, 25] [5, 6] [26]. A number of other misconceptions are common including beliefs that the condition is contagious or influenced by other environmental exposures (e.g., snake bites) [6, 25, 27, 28]. In turn, these inaccurate beliefs are associated with risk behaviors such as consistently walking barefoot [5, 6, 25] and holding stigmatizing attitudes towards patients [5, 6, 29, 30]. Whether engaging youth as disseminators of accurate GxE information could help to eliminate these misconceptions, reduce social stigma towards affected individuals and encourage preventive actions (i.e wearing shoes) is largely unexplored.

In order to consider the feasibility of engaging rural Ethiopian youth to serve as information disseminators in the spirit of LHWs, we posed four research questions: (1) what is the prevalence of youth misconceptions about the causes of podoconiosis?; (2) what factors are associated with accurate understanding of GxE contributions to podoconiosis among youth?; (3) is GxE accuracy associated with correct endorsement of preventive actions?; and (4) is GxE accuracy associated with confidence to explain the causes of podoconiosis?

## Methods

We conducted a cross-sectional survey in rural communities of the Wolayita zone of Southern Ethiopia with endemic podoconiosis [31]. Data were collected between August and September 2016. Two kebeles or lowest administrative units (Tome Gerira and Sura Koyo) were purposefully selected as neither had participated in prior studies of podoconiosis. Youth defined by United Nations as ages 15 to 24 were eligible to participate in the survey [32]. We used a random sampling strategy with all youth living in the area having an equal chance to be included in the sample. After conducting a census of youth in the participating kebeles 3,542 were identified in the target age range. 52% (n = 1,841) were females. Of youth identified, 11% (n = 383) were from families affected by podoconiosis. The optimal sample size required to detect differences of plus or minus five units in survey responses (with 95% confidence) was 347 [33]. We approached 377 youth in anticipation of 5% refusal to participate found in our prior studies.

Our prior qualitative findings suggested that there was a substantial knowledge disparity regarding podoconiosis causes and preventive actions between youth who were from affected families than youth who were unaffected [28]. Research to date has indicated that affected adults tended to endorse some explanations of the causes of podoconiosis more strongly than others [6]. Thus, we aimed to proportionally represent affected youth in the sample size. To this end, our sample comprised 41 affected youth and 336 unaffected youth randomly selected from the census sampling frame. This ratio of unaffected to affected youth is representative of the population in the study districts.

### Instrument and data collection

The questionnaire was initially developed in English, translated into Amharic and Wolayitigna (common dialect in the targeted kebeles), then checked for accuracy and back-translated to English. Interviewers who had previous experience in similar studies were recruited using informal networks. These individuals (N = 10) received three-days of training on the objectives of the study, items included in the survey questionnaire, and how to carry out the survey. The survey was pilot tested in two nearby rural kebels (Sodo Zuria and Damot Woydie woredas) with 30 youth to assess the adequacy of the instruments. These youth were not included in the main survey. Results of the pilot indicated a few limitations such as spelling or grammatical errors that resulted in respondents showing minor hesitation and request for clarification revisions were made.

Interviewers administered the survey to the selected youth at their homes. The expectation was that the interviews would take approximately 45 minutes. Written informed consent was obtained from all participants including thumbprints for those unable to sign. Consent from parents or guardians was obtained for those ages 15 to 18. The respondents were given exercise books and pens as compensation for participation. All aspects of this study were approved by the Addis Ababa University College of Health Science Institutional Review Board.

### Measures

Selection of survey measures assessed domains of knowledge, and self-efficacy related to conveying GxE information. These constructs of Social Cognitive Theory [34, 35], were based on prior literature on requisite competencies for LHWs to be effective [36] and on our extensive prior qualitative data collected with adults in these communities [28]. Demographic characteristics assessed included: gender, education level, and age. Additional measures of civic engagement and interactions with health extension workers also were assessed.

Three domains of knowledge were assessed:

GxE knowledge was based on four questions that were adapted to the causal factors contributing to podoconiosis [23, 25, 37]. Youth were asked to rate each statements as “true,” “false” or “don’t know” (e.g., “A person can inherit proneness to have their feet be irritated by the soil.”). These measures were dichotomized based on the median score (2.1). Youth who scored three and four were labeled as “mostly accurate” (1) and those who scored less than three were labeled as “mostly inaccurate” (0).

Knowledge about preventive actions was measured by rated agreement with a list of 10 potential preventive actions that included both accurate and inaccurate options (e.g., wearing protective shoes every day–accurate; taking vaccination–inaccurate). Each accurate response was assigned one point. The preventive action score was dichotomized due to skewedness, with zero representing inaccurate understanding (0–3) and one indicating mostly accurate understanding (4–10).

Causal misconceptions were based on 14 questions derived from prior research conducted with adults living in podoconiosis-endemic communities [26]. Misconceptions were assessed with two subscales, a “contagion score” based on responses to 11 questions assessing perception of podoconiosis as a contagious disease. A second subscale was labeled “other misconceptions” and included three questions related to beliefs about bacteria, poor nutrition and evil eye as causes of the disease. Each endorsed misperception was assigned ine point and then summed for a total score.

Confidence to explain (i.e., self-efficacy) was based on nine questions (e.g. “I am confident that I could explain to other people why some individuals develop podoconiosis and others do not”) with three response categories (1 = Disagree, 2 = Undecided, 3 = Agree) [26]. Scores were dichotomized as being above or below the median, where zero represented “less confidence” and one indicated “more confidence”.

Extracurricular civic engagement was based on self-reported involvement in extracurricular activities at school, youth association, Sunday schools and other leadership-oriented experiences.

In Ethiopia more than 30,000 health extension workers provide outreach services and disseminate health information to the general public to encourage health promoting habits (Federal Ministry of Health, 2007). Reported contact with health extension workers (HEWs) was assessed as they may have provided opportunities for youth to increase knowledge of health issues and podoconiosis, specifically. Youth who were visited by HEWs at their residence one year prior to the study were compared to those reporting no contact or visit.

### Data analysis

Data were analyzed using SPSS version 20 software. Frequencies and distributions were examined to check for out-of-range values and other errors in the data. After data cleaning, descriptive analyses, bivariate analyses and logistic regressions was performed. Cross tabulation and χ2 tests were performed for associations amongst variables. Level of statistical significance was set at p <0.05 (two-tailed). Variables with significant associations in cross-tabulation were entered into the logistic regression models to test in turn their association with accuracy of GxE knowledge, accuracy of endorsed preventive actions and confidence to explain GxE causes of podoconiosis. We ran Pearson’s correlation to assess the association between accurate GXE knowledge and prevalent misconceptions (i.e., inaccurate causal beliefs). Odds ratios were computed for the likelihood of having mostly accurate (1) and mostly inaccurate (0) understanding about GxE influences and appropriate preventive actions. Odds ratios were also computed for the likelihood of being more confident or less confident to explain podoconiosis-related information.

## Results

All 377 who were approached agreed to participate in the survey; 52% were female (See Table 1). The majority of youth participants were in the age range of 15–18 (78.5%). Accordingly, the majority of youth (70%) reported being in 7^th^ to 12^th^ grade education. Just over half reported involvement in some extracurricular civic engagement. Approximately 80% of the youth had contact with HEWs at least once in the year prior to the survey. Eleven percent of participants reported having a blood relative with podoconiosis.

**Table 1:** Characteristics of youth participants (N = 377).

Characteristic	n (%)
Education	
No formal education	19 (5)
Grades 1–6	81 (22)
Grades 7–12	264 (70)
College/Vocational school	13 (3)
Female gender	196 (52)
Age	
15–18	296 (78)
19–24	22 (81)
Have a family member with podoconiosis (affected)	41 (11)
Have friends with podoconiosis	44 (12)
Had had contact with health education worker	301 (80)
Involvement with extracurricular activities	221 (58)
Mostly correct GxE knowledge	168 (44)
Endorse contagion as cause of podoconiosis	189 (50)
Mostly correct Preventive Action knowledge	197 (52)
Confident to explain causes of podoconiosis	174 (46)

### How prevalent are misperceptions about the causes and prevention of podoconiosis among youth?

Half of youth believed that podoconiosis is a contagious disease and over one-third also endorsed several causal factors that constitute misconceptions including evil eye, and snake bites. Eleven percent of participants responded correctly to all four of the GxE knowledge questions related to podoconiosis, 34.5% answered three correctly, and 55% answered two or less questions correctly. The highest proportion of correct responses (82%) was agreement with the question “walking barefoot triggers podoconiosis among susceptible individuals” (See Table 2). Additionally, 61% agreed that susceptibility to podoconiosis is passed from generation to generation.

**Table 2:** Proportion with accurate responses to GxE knowledge questions (N = 377).

GxE Knowledge	n (%)*
A person can inherit proneness to have their feet be irritated by the soil (True)	219 (58)
Whether one’s feet become irritated by walking barefoot can be passed from one generation to another (True)	230 (61)
Individuals are born with susceptibility to podoconiosis that gets triggered by walking barefoot (True)	309 (82)
A person who has relatives with podoconiosis will certainly get the disease (False)	171 (45)
Mean GxE accuracy score (range 0–4)	2.4 (s.d. = 1.1)

*Percent accurate

A number of preventive actions were erroneously thought to be protective against podoconiosis, including vaccination (87%) and avoiding walking barefoot in cold weather or dew (87%). Additionally, one-third of youth incorrectly suggested avoiding personal contact with affected people, and nearly three-quarters endorsed avoiding wearing second-hand shoes, both indicators of belief in contagion. However, a majority of the youth also accurately endorsed regular foot washing (86%) and wearing protective shoes everyday (70%).

Due to their high co-prevalence, we tested the association of accurate GxE knowledge with prevalent misconceptions (e.g., endorsement of podoconiosis as a contagious disease). We found a modest positive correlation between GxE knowledge and contagion beliefs (r = 0.23, p<0.05). Youth with more accurate understanding of GxE contributors to podoconiosis also were more likely to harbor “other misconceptions” (r = 0.13, p<0.05).

### What factors are associated with accurate understanding of GxE contributors?

Participants with 7^th^ to 12^th^ grade education, who reported having affected family members or a friendship relationship with an affected individual were significantly more likely to be accurate in their understanding (correct responses to 3 of 4) of GxE contributors to podoconiosis than their peers (See Table 3). Endorsement of contagion as a cause of podoconiosis and interactions with HEWs also were significantly associated with accurate understanding of GxE contributors.

**Table 3:** Characteristics of youth and accuracy of GxE knowledge.

Characteristics		Percent with inaccurate GxE knowledge % (n)	Percent With Accurate GxE Knowledge % (n)	p-value
Age	15–18	54.4 (161)	45.6 (135)	0.70
	19–24	46.8 (46)	43.2 (35)	
Gender	Female	46.6 (111)	43.4 (85)	0.62
	Male	43 (96)	47 (85)	
Have a blood relative with podoconiosis	Yes (“affected”)	20 (8)	80 (33)	<0.001
	No	59.1(199)	40.9(137)	
Level of education	No-formal education	84.2 (16)	15.8 (3)	0.008
	Grade 1–6	89 (72)	11 (9)	
	Grade 7–12	54.5 (144)	45.5 (120)	
	College/vocational training	69.2 (9)	30.8 (4)	
Friendship with affected individuals	Yes	69.2 (9)	30.8 (4)	<0.001
	No	31.8 (14)	68.2 (30)	
Had contact with health education workers	Yes	54.9 (183)	45.1 (150)	<0.001
	No	49.2 (148)	50.8 (153)	
Civic engagement	Yes	77.6 (59)	22.4 (17)	0.07
	No	51.1 (113)	48.9 (108)	

Variables found to be significant in bivariate analyses were tested in multivariate logistic regression to predict accurate GxE knowledge (See Table 4). Affected status, having friendships with affected individuals, reporting any contact with HEWs, having some formal education and having a high contagion inaccuracy score were significantly associated with increased accuracy of GxE understanding.

**Table 4:** Multivariable logistic regression models testing factors associated with accuracy of GxE, actions to prevent knowledge, and confidence to explain causes of podoconiosis.

Independent variables	Accuracy of GxE knowledge		Accuracy Preventive Actions Knowledge		Confidence to Explain	
	OR	p-value	OR	p-value	OR	p-value
Constant	0.16	0.000	0.28	0.050	0.09	0.000
Education 1–6 grade (vs. no-formal education)	11.8	0.003	1.1	0.91	0.77	0.64
Education 7–12 grade (vs. no-formal education)	8.7	0.008	1.8	0.28	1.11	0.83
Education–College/Vocational (vs. no-formal education)	4.8	0.12	3.1	0.16	1.31	0.72
Affected by podoconiosis (vs. non-affected)	8.0	0.0001	2.1	0.08	0.98	0.05
Friendship with affected individuals (vs. not having friendship)	3.0	0.009	2.4	0.03	1.75	0.13
Had contact with HEW in past year (vs. no-contact with HEW)	3.8	0.0001	3.8	0.0001	1.42	0.24
Endorse contagion as cause of podoconiosis (vs. not endorse contagion as cause of podoconiosis)	1.18	0.001	0.92	0.02	1.05	0.20
Accurate GxE knowledge	—	—	1.01	0.93	1.30	0.03
Accurate preventive actions knowledge	—	—	—	—	1.28	0.0001
	Model χ2 = 439.77, p<0.05		Model χ2 = 467.61, p<0.05		Model χ2 = 487.80, p<0.05	

Youth who had attended formal schooling from grade 1^st^ to 6^th^ or 7^th^ to 12^th^ education level had 11.8 and 8.7 times, respectively (95% CIs: 2.2–16.6; 1.7–13.4) the odds of having accurate GxE knowledge related to podoconiosis as those without formal schooling. Affected youth had 8.0 times the odds of being accurate in GxE knowledge as unaffected youth (95% CI: 2.9–16.1). Similarly, youth with affected friends had three times the odds of having accurate understanding as those who did not report having friends with podoconiosis (95% CI: 1.3–6.9). Youth who reported contact with HEWs had 3.8 times (95% CI: 2.0–7.2) the odds of knowing about the joint contribution of GxE in the development of podoconiosis as those who did not report contact with HEWs. Youth with stronger contagion beliefs had 1.2 (95% CI 1.0–1.2) times the odds of holding accurate GxE knowledge as those with weaker contagion beliefs.

### Is GxE accuracy associated with correct endorsement of preventive actions?

We tested a logistic model that included the factors found above to be associated with accuracy of understanding in a model for appropriate endorsement of preventive actions (dichotomized as 0 (1–3) = mostly inaccurate and 1(4–10) = mostly accurate). Results showed that youth who reported having a friendship with an affected individual had 2.4 times (95% CI: 1.0–5.4) the odds of of endorsing appropriate preventive actions as those without affected friends (See Table 4). Youth who reported contact with HEWs had 3.8 times (95% CI: 2.0–7.0) the odds of endorsing appropriate preventive mechanisms as those who did not report contact with HEWs. Youth who endorsed contagion as a cause of podoconiosis had lower odds of accurately endorsing preventive actions as those who did not endorse contagion beliefs (OR = 0.92, 95% CI: 0.8–1.0). Counter to our expectation, accurate understanding about GxE contributors to podoconiosis was not associated with endorsement of appropriate preventive actions (e.g., shoe wearing, p = 0.92).

### Is GxE accuracy and endorsement of appropriate preventive action associated with confidence to explain the causes of podoconiosis?

We tested a logistic model that included the factors found above to be associated with appropriate endorsement of preventive action to test their association with confidence to explain the causes of podoconiosis to others (dichotomized as 0 (1–3) = less confidence, 1 (4–10) = more confidence). Results showed that those affected by podoconiosis had lower odds of being confident to explain GxE influences as unaffected youth (OR = .98; 95% CI: 0.1–1). Accuracy of GxE and preventive action knowledge were significantly associated with confidence to explain the causes of podoconiosis (See Table 4). For each unit increase in GxE accuracy and endorsement of preventive action, the odds of being confident increased by a factor of 1.3 (95% CI: 1.0–1.7) and 1.3 (95% CI: 1.1–1.5) respectively, when compared to the reference group.

## Discussion

Our findings show that youth in podoconiosis-endemic communities have similar misconceptions as those reported previously among adults in neighboring communities [4, 6, 25, 38]. Like adults, youth endorsed multiple causes of podoconiosis that included both contagion and environmental exposures (e.g., snake bite, evil eye). However, a sizable proportion of youth responded accurately to questions about the joint contributions of heredity and environment in the development of podoconiosis. It is concerning that youth with the most accurate understanding of GxE contributors to podoconiosis also were most likely to harbor misconceptions about the causes of podoconiosis. Thus, endorsement of GxE contributors did not obviate other erroneous causes such as contagion.

Our previous qualitative work with youth in these communities that aimed to understand youth’s explanatory mental models documented a similar connection between GxE and contagion beliefs [28]. In our previous report we posited that beliefs that susceptibility to podoconiosis passes from generation to generation (i.e., accurate GxE knowledge) co-occurred with observations of families living in close proximity to each other where bodily fluids could be exchanged. Thus, youth perceived a “both/and” connection in which inherited susceptibility and contagion explanations could co-exist.

Generally, health education programs assume that accurate health information can correct inaccurate beliefs. However, our findings suggest that community members can hold numerous, even contradictory, notions of causation simultaneously. The implications of this present challenges for engaging youth as LHWs. Contagion beliefs not only shape priorities given to preventive actions but also fuel stigmatizing beliefs. Endorsement of both GxE and contagion as causes of podoconiosis also may have attenuated the association between related knowledge and endorsement of appropriate preventive behaviors. Thus, any efforts to engage youth as LHAs to promote preventive actions among affected individuals would also need to challenge beliefs about contagion.

A large body of literature has shown that contagion beliefs have deep roots and are very difficult to override [28, 39]. In this study, youth who reported having social ties to affected individuals were more likely to be accurate in their understanding of GxE contributors. Similarly in our prior intervention study, affected adults were less likely to endorse contagion beliefs than unaffected adults [26]. Individuals with social ties to affected individuals, particularly those who have engaged with health extension workers may be more accepting of alternative causal explanations that are linked to preventive actions.

Level of education was also associated with GxE knowledge accuracy. Youth without formal education had lower knowledge about podoconiosis, which is consistent with earlier studies among adults [24, 40, 41]. Lack of schooling limits youth’s access to health information provided in school curriculums and extracurricular activities. Youth engaged in formal education could be optimal disseminators of accurate GxE information. Indeed, prior studies identified some schooling to be a crucial qualification for LHWs in Peru and the Republic of the Congo [42, 43]. However, education level was not associated with accurate knowledge of preventive actions or with confidence to explain in this sample. LHA training targeted to school settings would need to strengthen these skills among youth.

The study further showed that accurate knowledge about GxE contributors of podoconiosis may not necessarily lead to understanding accurate preventive actions. Experts argue that individuals’ understandings of causes of health conditions have strong influences on what they perceive as best preventive actions to take to lower risk of developing diseases [44, 45]. The logical assumption is that provided with accurate causes of health conditions, individuals will be more likely to identify preventive actions to lower the risk of developing ailments. The concern is that holding a number of competing beliefs about the causes of podoconiosis could muddle youth’s understanding about what to do to prevent the conditions.

LHWs training should not assume that improved understanding and acceptance of GxE causal mechanisms will override beliefs about contagion. Curricula specifying the mechanism through which soil exposure (e.g., silica particles) leads to lymphatic inflammation could be included in school science classes. In this environment, classroom exercises could be used to encourage youth to consider these mechanisms and debate the other causal beliefs using peer discussions and co-teaching.

It is heartening that youth with higher GxE accuracy and preventive knowledge also had the highest confidence in their ability to explain the causes of podoconiosis. It has been suggested that effective LHWs have conceptual understanding and confidence to effectively communicate with their constituencies that, in turn, can be capitalized upon to facilitate individual- and community-level health promotion [36]. Similarly, a recent study found confidence to talk about breast and cervical cancer screening information to significant others to be an important individual characteristic for LHWs [36]. However, affected youth had lower self-confidence, despite their greater GxE knowledge compared to unaffected youth. This suggests that confidence-building strategies may need to be different for affected and unaffected youth. Stigma related to being affected by podoconiosis may have lowered self-esteem of these youth and inhibit their confidence to discuss the topic. It also should be noted that further assessment might be needed to identify the factors that motivate young people, both those affected and unaffected, to take part in podoconiosis prevention campaigns.

The study had several noteworthy strengths including a high participation rate, and the survey development was informed by extensive qualitative data collection. However, there were a few limitations. The study was conducted in only two rural communities that may not be generalizable to youth in urban areas or other LMIC settings. The survey focused specifically on factors associated with youth’s accuracy of knowledge of podoconiosis causes, GxE influences and preventive actions with the aim of informing health literacy-building activities. Numerous other factors could influence the viability of youth in these settings serving as LHWs. Indeed, studies show that role-related factors, social network and trust, and other characteristics of target communities can influence motivation and performance of LHWs that were not assessed [11, 36, 46, 47]. Moreover, knowledge improvements unto themselves may be necessary for health promotion but are unlikely to be sufficient to influence behavior change. Our findings of coincident endorsement of causal beliefs related to GxE and contagion may have been influenced by social desirability bias, with participants eager to endorse multiple causes and inherently multiple solutions to the problem.

These results are preliminary but support the pursuit of further research to consider youth assets and deficits relating to their potential role as LHWs. Additionally, extensive qualitative and quantitative studies will be required to guide identification of youth who may be most willing and competent in this role and to evaluate interventions to help youth acquire skills and assess community impact. The potential to avoid disparities in reach of emerging genomic knowledge warrants these efforts.

## Supporting information

S1 Strobe checklist (DOC)

S1 Dataset (SAV)
